# Connecting Scientists Residing Abroad: A Review of *Converciencia* as a Practice to Engage the Guatemalan Scientific Diaspora From 2005–2020

**DOI:** 10.3389/frma.2022.898496

**Published:** 2022-06-27

**Authors:** Kleinsy Bonilla, Susana Arrechea, Luis Guillermo Velásquez Pérez

**Affiliations:** ^1^Department of Science and Technology Policies (DPCT), Institute of Geosciences (IG), State University of Campinas, Campinas, Brazil; ^2^Instituto Para el Desarrollo de la Educación Superior en Guatemala (INDESGUA), Guatemala City, Guatemala; ^3^New Sun Road, P.B.C., Richmond, CA, United States

**Keywords:** science diasporas, S&T policy, S&T capacity building, Guatemala, *Converciencia*, brain drain-brain circulation, knowledge network diasporas, skilled migration

## Abstract

In 2005, the Guatemala National Secretariat of Science and Technology (Senacyt) introduced *Converciencia*, a program designed to connect Guatemalan scientists residing abroad with their country of origin. *Converciencia* has been a flagship practice for over 15 years. This program involves three main groups of participants: (i) science and technology (S&T) policy agents, (ii) the scientific community (including parts of the Guatemala scientific diaspora, GSD), and (iii) host institutions (local co-organizers, mainly universities, and research institutes). This article presents a comprehensive and balanced overview of the *Converciencia* program applying an in-depth analysis of its creation, evolution, leading trends, and legacies. Using a qualitative methodology and conducting a four-level analysis (descriptive, explanatory, normative, and prescriptive) allowed for the identification of nuances of this S&T practice in the context of a scientifically lagging country such as Guatemala. The detailed data collected through documentary and desk review, gray literature, focus group discussions, and semi-structured interviews resulted in a framework to highlight the strengths, weaknesses, opportunities, and threats (SWOTs) in the planning, organization, implementation, monitoring, and perception of the results achieved by *Converciencia*. Findings portray the contrasting views and perceptions from a single S&T practice, depending on the participating parties' roles and responsibilities. Direct participants examined how *Converciencia* has achieved its objectives while questioning the effectiveness and impact that the resources allocated to the initiative have yielded over time. Evidence indicates that despite the design, coordination, and evaluation limits of *Converciencia*, the GSD, the scientific community in Guatemala, and the host institutions are interested in the continuity of the practice. Indeed, the main recommendation involves restructuring and turning *Converciencia* into a robust S&T policy. *Converciencia* as a policy engaging the GSD could produce greater results and impacts by involving all the key actors in co-designing activities, clearly determining roles and responsibilities, and establishing performance and impact indicators for evaluation.

## Introduction

The emigration of talented and highly educated individuals from developing to advanced countries has been of interest in literature for several decades (Meyer and Brown, 1999; Commander et al., 2004; Lewin and Zhong, 2013). Although the dominant paradigm has been brain drain (Commander et al., 2004), a shift in the narrative proposes a new approach toward brain circulation and the power of knowledge networks to enable those individuals to remain relevant to their country of origin (Meyer and Brown, 1999). Various studies have focused on the diaspora and how countries develop policies with their populations abroad (Davenport, 2004; Ragazzi, 2014). Different geographic contexts have explored the topic in their territories, such as China, India, and Russia (Li et al., 2019; Yurevich et al., 2019). However, limited research has addressed the emergence and engagement of scientific diaspora (SD) in the Latin America region. A recent study on the role of skilled migration in the Caribbean (Alleyne and Solan, 2019) has concluded that it is likely that skilled migration will continue and that developing countries must seek to benefit from such migration. The study also argues that diaspora networks can help stem the long-run migration of highly skilled workers, but this will depend on improving local conditions for those at home and those who may wish to return or collaborate through networks. In the last decade, “brain circulation” has taken further relevance *vis a vis* the traditional ideas of brain drain because of the growing mobility of talents across international boundaries (Tung, 2008). Guatemala requires a stable, effective, and long-term national S&T policy with valuable cooperation provided by the SD, partner countries, and international higher education institutions to turn brain drain into brain circulation (Bonilla et al., 2018). Other researchers have also referred to brain networking as a feasible methodology given the difficulties of reversing brain drain and creating brain circulation in developing countries (Ciumasu, 2010). The study of Ciumasu identified policy preference and implications for the systematic development of linkages between SDs and resident scientists creating brain networking. Valuable progress has been made in defining the problem and formulation of science, technology, and innovation. Still, the scope of the concepts concerning developing countries in the Central American region in the global context is pending (Viales-Hurtado et al., 2021). Expansion of the research agenda would strengthen the SD community's ability to inform policies for societal change, considering the “innate interest of the research community in innovation and development in activating change through public policy” (Lema et al., 2021, p. 1). There is an added value of network dependencies capturing political and cooperative interactions across countries (Kammerer and Namhata, 2018). The research by Kammerer and Namhata (2018) finds that adoption of climate policies is a matter of social influence. Governments are more likely to adopt policies if they cooperate with countries that have adopted more climate policies and are in a similar structural position to countries active in climate protection. Therefore, a similar approach could be taken to other science and development policies with the influence of the SD. In other words, public policies and practices engaging the GSD, including interactions across countries, are vital for contributing to the scientific communities in Guatemala.

From the policy perspective, some cases can be highlighted in the context of Latin America, particularly the cases of Colombia, Argentina, Mexico, Brazil, Uruguay, and Costa Rica. Chaparro et al. (2016) show that in Colombia, the Caldas Network is a case worthy of analysis, as it is a pioneer in the region. This initiative was created as *Red Colombiana de Investigadores en el Exterior R-Caldas* (Colombian Network of Researchers Abroad). It was founded in 1991 and managed by *Colciencias* (formerly Administrative Department of Science in Colombia, which is currently part of the Ministry of Sciences). Its purpose was to channel the research potential of Colombians living abroad to the country and link them to research institutions for the creation of scientific capacity. *R-Caldas* relied on connections among Colombian diplomatic missions abroad to identify and reach out to the Colombian SD. The engagement activities of the members of the *R-Caldas* were classified into four categories: (i) identification of research areas with possibilities of cooperation and formulation of joint projects, (ii) training of human resources and return of researchers, (iii) internships and mobility, and (iv) communication and scientific dissemination. In Argentina, *Programa Raices* (a network of Argentine researchers and scientists) was designed by the Ministry of Science, Technology, and Productive Innovation in 2003. It became a national policy in 2008 managed by the National Agency for Scientific and Technological Promotion and the National Council for Scientific and Technical Research. The objectives included permanent or temporary repatriation of Argentine researchers residing abroad, development of networks linking local researchers and those of the diaspora by realization of short stays, dissemination of job opportunities agreed upon with the private sector and foundations, and dissemination of scientific and technological activities (Charreau, 2011; Luchilo, 2018). The program is still actively pursuing four lines of action: (i) acknowledgment and repatriation, (ii) strengthening of research capacities in Argentina, (iii) engaging Argentinian scientists residing abroad, and (iv) sharing opportunities for career progression. In Mexico, the program *Red de Talento Mexicano* started in 2000 under the Network of Mexican Talents Abroad as a result of spontaneous actions from individuals who later received official support from the Government of Mexico. Since 2013, the network has been known as Red Global MX. Currently, it is managed by the Ministry of Foreign Affairs and the National Council for Science and Technology. Initially, it was focused on Mexican scientists residing in the United States of America to promote the integration of Mexico into the global knowledge economy through scientific dissemination events, academic cooperation, and linkage projects with strategic sectors. Currently, Red Global MX has a portfolio of more than 180 projects that link its members directly with Mexico and help promote the name of Mexico abroad. It is organized through local chapters located in four regions worldwide: the United States and Latin America, Canada, Asia-Oceania, and Europe. It consists of 58 chapters in 28 countries and has more than 6,500 members (de la Barrera Soria, 2011; Red de Talentos Mexicanos, 2020). In the case of Brazil, Carneiro et al. (2020) analyze the case of *Rede Diáspora Brasil* (Brazilian Diaspora Network), a project developed between 2013 and 2016, as a series of engagement strategies related to the Brazilian diaspora of science, technology, and innovation promoted by the Brazilian government. This project was designed and implemented by the Brazilian Industrial Development Agency (Abdi) and was funded by the Brazilian Trade and Investment Promotion Agency (Apex-Brasil). The project aimed to set up a “network of networks,” engaging active initiatives and Brazilian professionals (executives, entrepreneurs, and scholars) and foreign professionals with ties with Brazil. Some of the project's main components included building partnerships and research cooperation to develop business and projects in intensive knowledge and technology areas. The case of Uruguay involves the program *Circulación de Uruguayos Altamente Calificados* (Circulation Program for Highly Qualified Uruguayans, CUAC). The program was created in 2005 to “actively and effectively link highly qualified Uruguayans residing abroad with national institutions” (Ministerio de Relaciones Exteriores, 2022, p. 1). This was sought primarily through innovation projects and the promotion of national capacities and knowledge transfer (Taks, 2006). Finally, in the Central American region, Costa Rica presents the case of *Hipatia* (Santos Pasamontes, 2022). This program was created in 2014 and managed by the National Council of Rectors to provide an updated overview of the country's science, technology, and innovation capabilities to support public-private decision-making. Though this program, by bringing together the supply and demand for technology and qualified human resources through the *Hipatia* portal, professionals in the diaspora have been identified in more than twenty science and technology (S&T) disciplines. The diversity would facilitate communication between colleagues to implement collaboration mechanisms in multiple fields. Several of them intersect with aspects related to the country's development process.

In the case of Guatemala, there are no studies focused on the engagement of the Guatemala Scientific Diaspora (GSD) or significant policies addressing the relevance of the migration of skilled individuals and the eventual connection among them and with their country of origin. This research intends to bridge such gaps. This article presents a comprehensive review of *Converciencia* as the single identified initiative designed and implemented by the Guatemala National Secretariat of Science and Technology (Senacyt) aimed at connecting Guatemalan scientists living and working abroad with their country of origin. This initiative has incorporated the participation of grassroots actors, including members of the GSD and host institutions (co-organizers). *Converciencia* has also mobilized broader sectors of the society, such as the media, the industry/productive sector, other public institutions, organizations from the civil society, and students in middle, high school, and universities. *Converciencia* has used valuable and scarce human and financial resources, which should be analyzed against the achieved results. The review of this S&T practice follows a four-level analysis adapted from Patton et al. (2013, p. 31) that is: (i) descriptive, (ii) explanatory, (iii) normative, and (iv) prescriptive. In addition, the SWOT (strengths, weaknesses, opportunities, and threats) method was applied to enhance the discussion while incorporating the perspectives of the parties directly involved in the organization/implementation of *Converciencia* each year. The SWOT method has been historically used as an organizational tool for strategic planning (Gurel and Tat, 2017). This tool allowed for highlighting the limitations reported by stakeholders and participants. Finally, as part of the prescriptive analysis, participants also propose actionable recommendations to improve *Converciencia*. This article provides readers with the first comprehensive overview of activities, involved actors and stakeholders, resources invested, results, and lessons from *Converciencia* as an S&T practice to engage the GSD for over 15 years.

## Methods

This study was carried out using a qualitative methodology in which three types of methods were used to collect data: (i) focus group discussions, (ii) semi-structured interviews with key respondents, and (iii) documentary and gray literature review. Primary and secondary data were collected to incorporate the perspectives of the three main parties involved in *Converciencia* since its origin.

Table 1 summarizes the participants in the study who provided data from the policy perspective. The first group comprises all individuals who have occupied the National Secretary of Science and Technology position in Guatemala during the study period (Table 1).

**Table 1:** Participants from the policy perspective: secretaries of science and technology in Guatemala (2005–2020).

**Secretary**	**Period**	**Profile summary (education and occupation)**
Rosa María Fabian Amaya	2005–2008	Economist—Usac (Guatemala), Master in Operations Research Ufm (Guatemala), Doctor in Research Sciences (Umg (Guatemala)/Professor at Usac School of Economic Sciences
	2009–2012	
Miriam Rubio Contreras	2013–2014	Industrial Engineer—Usac (Guatemala). Institutional Projects Advisor to the Rector, Usac Consultant and Technical Advisor Intecap. Professor, Usac
Armando PokusYanquián	2014–2016	Civil Engineer from Usac and Project Manager and magister from Umg. Investment project management consultant with experience as Professor from local universities
Oscar Cobar Pinto	2016–2019	Pharmaceutical Chemist from Usac, Doctor in Chemistry specializing in Organic Chemistry, University of Puerto Rico. Usac Professor and Former Dean of the Faculty of Chemical Science and Pharmacy, Former Director of Research DIGI Usac
Ana Judith Chan	2020–incumbent	Lawyer, Master's in International Business Law from the *Pontificia Universidad Católica de Chile*. Former President Guatemalan Mexican Chamber of Commerce and Industry (Camex)

*Source: based on public records and available public information from the participants. Usac, San Carlos of Guatemala University; Ufm, Francisco Marroquin University; Umg, Mariano Galvez University; Intecap, Institute of Technical Training and Productivity; DIGI, Directorate of Research*.

The second group of participants provided a perspective from the Guatemala scientific community, mainly members of the GSD who have participated in one or more editions of *Converciencia*. This category emphasized the selection criteria (see Tables 2, 3). Diverse representation was sought in terms of gender balance, fields of knowledge, stages in their career, and the country/location of the destination. From semi-structured interviews, nearly 30 h of audio material was collected from the application of semi-structured interviews and focus group discussions. Each interview session had an average duration of 45 min, while the focus group discussions lasted for 60 min.

**Table 2:** Criteria for participant selection: focus group discussions, Guatemala scientific community.

**Criteria**	**Description**	**Operationalization**
Experience	Experience in community building or participation, networking, groups of scientists	Reporting experience in building and/or participating
Trajectory	Procure diversity in the representation of career development stages of the interviewees (early and mid-established career).	Years since completion of graduate studies. Early <10 years, Mid +10 years but no management positions or group coordinators. Established +15 years in addition to management or research group coordination positions
Field of expertise	Diverse fields of knowledge (i.e., natural sciences, health, earth science, social sciences, physics, and engineering sciences)	All fields of knowledge were considered, including social, natural, and engineering sciences
Destination diversity	Covering a wide range of geographic locations for destinations.	Including as many geographic destinations as possible region/country, i.e., North America, Europe, Asia, and Latin America
Gender balanced	Balanced participation of women and men	Gender equality in participation.

**Table 3:** Participants from the scientific community, Guatemala scientific diaspora (GSD): two focus group discussions.

**Stage in career**	**Country of residence or experience as GSD**	**Gender balance**	**Research area**
**Group A: scope: pro-** ***Converciencia***			
Established career	Chile	M	Electronic engineering/wireless communications
Early-Career	Taiwan	F	Biomedicine/nanotechnology
Mid-Career	Sweden	F	Water studies/limnology
Established career	Spain	F	Health sciences/virology
Mid-Career	United States	M	Mathematics/mathematical physics
Established career	United States	M	Physics/gravitation and numerical relativity
Established career	Germany	F	Environmental sciences/water bioindicators
**Group B: scope: critic:** ***Converciencia***			
Mid-Career	Chile	M	Social sciences/science communication
Early career	Costa Rica	F	Biology/endemic species and conservation
Established career	Spain	M	Molecular biology/biotechnology
Established career	Spain	F	Chemical sciences/toxicology
Mid-Career	United States	F	Medical and health sciences/neurosciences
Established career	Canada	F	Food and nutritional sciences/iron safety studies
Established career	Germany	F	Public health nutrition/meal patterns in school children

*N = 14, F, Female (N = 9); M, Male (N = 5)*.

From a preliminary list of 40 potential key respondents, a total of 14 participated in two focus group discussions.

As for host institutions, five co-organizing partners of *Converciencia* were selected based on their relevance to the program's science and research capacity-building component. Table 4 below summarizes the selected participants.

**Table 4:** Selected host institutions: perspective for local partnerships for capacity building.

**Code for references**	**Institution**	**Department**	**Relevance**
Host Institution 1	Universidad de San Carlos de Guatemala Usac	Faculty of Chemical Sciences and Pharmacy Faculty of Engineering	Usac is the only public University in the country. It is responsible for directing, organizing, and developing higher education in Guatemala One of its missions is to promote research in all spheres of human knowledge and cooperate with actors in the national innovation system to produce responses to national needs The academic units included in the study have participated in various editions of *Converciencia* A significant group of the GSD has obtained their undergraduate degrees in this University from these academic units. The Faculty of Chemical Sciences and Pharmacy designed and ran a joint doctorate program initiated through *Converciencia* between Unam and Usac
Host Institution 2	Universidad del Valle de Guatemala Uvg	Directorate of Research *Centro de EstudiosAtitlán CEA*	Uvg is the leading private University in Central America and Panama by QS Ranking 2022 This University employs prominent local established researchers who have partnered with *Converciencia* Uvg participated for several years as a *Converciencia* host institution A significant group of members of the GSD had obtained their undergraduate degrees from Uvg and kept collaborating with local researchers at Uvg
Host Institution 3	Universidad Galileo Ugal	School of Electronic Engineering	Ugal participated for several years as a *Converciencia* host institution Ugal has achieved research cooperation through **Converciencia*, resulting* in international publications
Host Institution 4	Universidad Rafael Landivar Url	Incyt Institute of Science and Technology Iarna Institute of Agriculture, Natural Resources and Environment	Url participated as a *Converciencia* host institution in various editions The Guatemalan Institute of Agriculture, Natural Resources and Environment of the Rafael Landivar University (IARNA—Url) has generated crucial grassroots research in critical fields for the country
Host Institution 5	Universidad Mariano Gálvez Umg	School of Medicine Laboratory of Genetic Sequencing	Umg has participated in various editions as a *Converciencia* host institution Umg has recognized research capacity through its laboratories with above average (in the national context) research infrastructure and scientific human power

*Source: based on interviews with staff and co-organizers from the host institutions, complemented with publicly available information. Usac, San Carlos of Guatemala University; Unam, National University Autonomous of Mexico; Url, Rafael Landivar University; Umg, Mariano Gálvez University*.

In addition, the authors developed a body of gray literature for review using annual reports from Senacyt from 2005 to 2020. This was complemented by media coverage and published news related to *Converciencia* during the period of the study. In addition, we reviewed the public records of the meetings from the International Network of Science, Technology, and Innovation (*RedCTI*). The review of relevant literature contributes to a deeper understanding of the policy and practice analysis. Complete details of the participants can be found in the supplementary data. Following data collection, a multi-layered policy analysis was conducted.

## Results and Discussion

This section presents the discussion and findings following a four-level policy and practice analysis. Table 5 summarizes descriptors, questions for operationalization, and perspectives in each level of analysis.

**Table 5:** Levels of analysis, *Converciencia*: policy and practice review.

**Level of analysis**	**Descriptor**	**Operationalization—questions to answer**	**Perspective**
Descriptive	It refers to the narration of the general characteristics of the policy/program/practice. Includes the most relevant features including origin, vision, participants, and resources	How was the policy/program/practice created? Who are the participants? How do actors participate? What does the policy/program/practice consist of? Which are the general characteristics?	S&T Policy Agents Media Coverage
Explanatory	It refers to the argumentation regarding the elements that impact or cause the analyzed policy/program/practice	Why was the policy/program/practice created? Why do participants take part in it? What do they do it for? What are their motivations and expectations?	S&T Policy Agents, Scientific Community, Host Institution
Normative	It refers to the set of ideas and the worldview held by the actors that influence (justify) their behavior, actions, and decisions. It is largely based on their interpretation of the ideas that shape the policy/program/practice under analysis	What are the perspectives on the general topic? What are the perspectives on the specific topic? What are the problems with the situation?	S&T Policy Agents, Scientific Community, Host Institution
Prescriptive	It refers to the “ideal” version of the policy/program/practice, a set of recommendations	What should the ideal situation look like? What should happen to improve the practice? What are the expectations? Which are the solutions to the identified problems with the practice?	S&T Policy Agents, Scientific Community, Host Institution

*Source: adapted from Patton et al. (2013, p. 31)*.

### Descriptive Analysis

This level of analysis refers to the general characteristics of the practice. It includes the most relevant features, namely, vision, parties involved, roles and responsibilities, and resources. It is essential to include the perspectives reported by all the parties involved.

**Figure 1:**
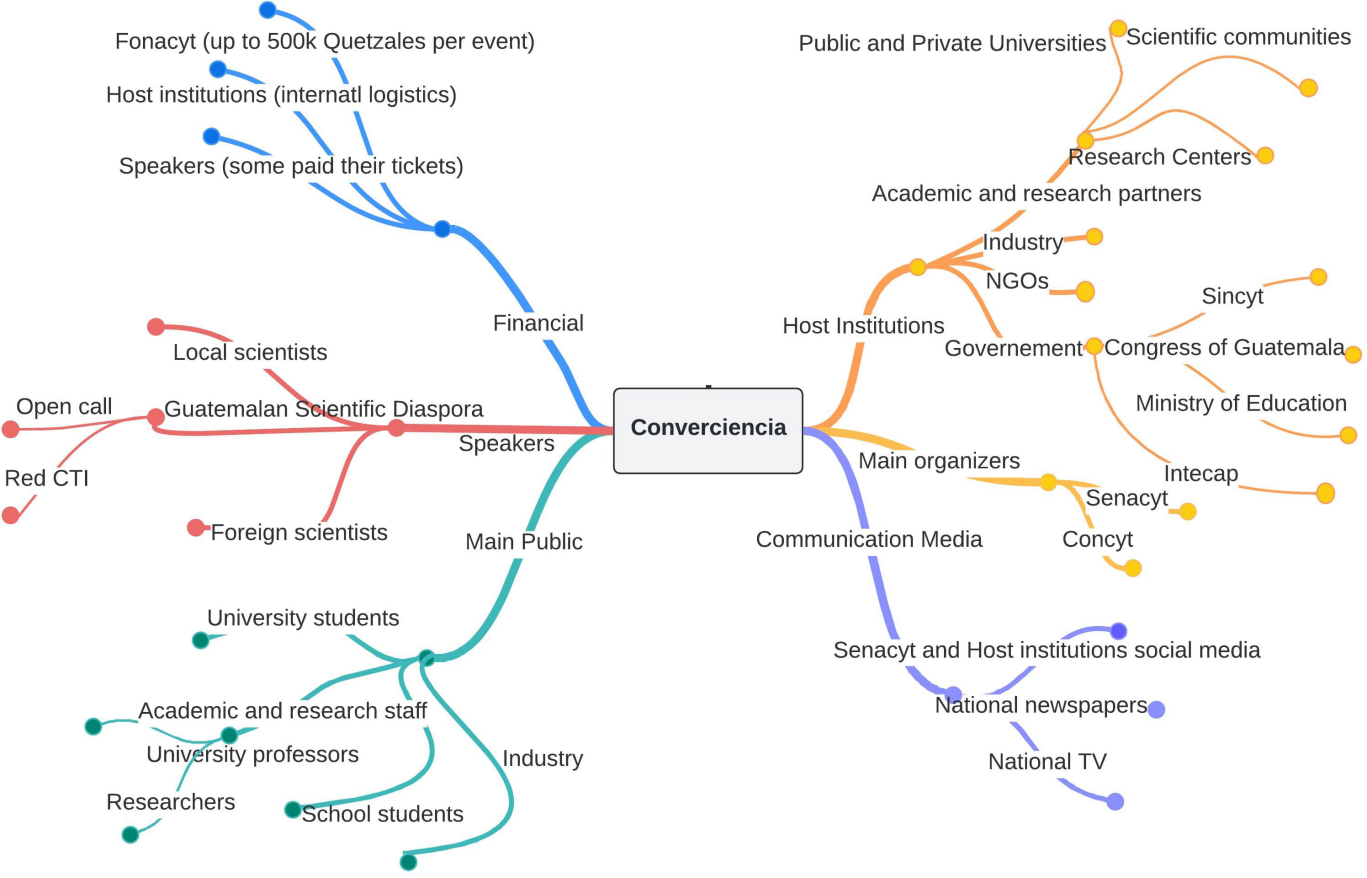
Actors in *Converciencia*: descriptive analysis. Source: own elaboration.

*Converciencia* as an S&T practice has taken place for over 15 years. One of its main objectives has been to connect the GSD with local scientists, the industrial sector, the academic community, and society. Institutions from the public sector involved in organizing *Converciencia* include the National Council for Science and Technology (Concyt) and Senacyt. Other institutions with indirect involvement in different editions have included the Ministry of Education, the Congress of Guatemala, and the Technical Training and Productivity Institute (Intecap). From the scientific community, the main representation has been the *RedCTI*. However, researchers and scientists working in local universities and research centers have also been part of *Converciencia* activities. Host institutions co-organizing *Converciencia* have mainly included those with presence in the capital city of Guatemala. However, in various editions, host institutions have participated in co-organizing/hosting *Converciencia* in a few departments (provinces) of Guatemala. Other actors with sporadic participation include international organizations, non-governmental organizations, and industry and private firms. In this level of analysis, it is crucial to describe the involvement of the main actors and stakeholders that have consistently participated in *Converciencia*. Figure 1 illustrates the diversity of actors and resources that this S&T practice has mobilized over time. The variety of actors mobilized and resources used in the context of *Converciencia* are illustrated in different colors, nodes, and veins.

#### The National Secretariat for Science, Technology, and Innovation (Senacyt)

Concyt formulates the country's science, technology, and innovation policy. It aims to direct and coordinate the national scientific and technological development with the executing functions of Senacyt (Jarquin-Solis and Mauduit, 2021). Concyt is chaired by the Vice President of Guatemala and has nine members: the Minister of Economy, the President of the Commission of Science and Technology from the Guatemalan Congress, one representative of the Chamber of Industry, one representative of the Chamber of Agribusiness, one representative of the Chamber of Commerce, the Rector of the San Carlos of Guatemala University (Usac), one Rector representing the private local universities, and the President of the Academy of Medical, Physical, and Natural Sciences of Guatemala. Senacyt executes and implements the policies and decisions of Concyt and constitutes the link between institutions that form the National System of Science and Technology (Sincyt). The Decree 63–91 Law created Sincyt for the Promotion of National Scientific and Technological Development, which constitutes the general framework for orienting scientific and technological activities in the Republic of Guatemala.^1^

Senacyt is the government institution that, in response to a mandate by Concyt, designed *Converciencia* and has kept the role of principal organizer of the annual event since its creation in 2005. A comprehensive review of all the available annual reports issued by Senacyt in the study period (2005–2020) provides a detailed summary of the main highlights of *Converciencia* reported each year. Table 6 summarizes the number of times *Converciencia* is referred to each year. Interestingly, some patterns emerge in terms of the absence of performance indicators and difficulties in conducting a comparative/monitoring analysis. Also, a period with no activities related to *Converciencia* was confirmed from 2013 to 2016.

**Table 6:** Descriptive analysis: detailed review of the annual reports, Senacyt (2005–2020).

**Year**	**Secretary senacyt**	**References to *Converciencia*—Highlights from the annual reports Senacyt 2005–2020**
2005	Rosa Maria Amaya	No annual report is available. Various consultations were carried out. Officers from Senacyt indicated there are no records of the 2005 Annual Report. However, *Converciencia* first edition dates to 2005 (SENACYT, 2022a,b), which is also reported in the Annual Report 2006
2006	Rosa Maria Amaya	*Converciencia* is reported in four paragraphs on page 21 (37 pages). Main highlights: Second consecutive edition of the event. Objective: Share the work of Guatemalan researchers residing abroad and promote an exchange with their peers residing in Guatemala. Main results: 40 leading conferences offered by the guest visiting researchers, estimated participation of 5,000 people in the different activities, and the celebration of the first International Assembly *RedCTI*. The term *Converciencia* is used a total of four times in the report
2007	Rosa María Amaya	*Converciencia* is reported on three pages, 30–41 (81 pages). Main highlights: 21 guests visiting researchers. Objectives: Foster interest and encourage young high school and college students toward research and science activities; involve different sectors (academic, private, and public) and the general public; raise awareness of the need and urgency to develop science and research for the development of Guatemala; increase the visibility of the work done by Guatemalan scientists visiting from abroad; and promote an exchange of visiting scientists with their peers residing in the country. No mention of outcomes of results. The term *Converciencia* is used a total of 18 times in the report
2008	Rosa María Amaya	*Converciencia* is reported on three pages 35–37 (77 pages). Main highlights: 17 Guatemalan scientists visited from abroad, and an estimated 4,000 people participated in *Converciencia* 2008. Strengthening the *RedCTI*. Objectives: Produce a critical analysis since the beginning of the event, formulation of innovations, improvements, and new academic approaches; the compilation of practical proposals to address the national crisis affecting the STI sectors; the establishment of new cooperation programs between local and foreign scientists and universities; the formulation of recommendations to the educational sector for the training of human resources in CTI. The term *Converciencia* is used a total of 13 times in the report
2009	Rosa María Amaya	*Converciencia* is reported on three pages 41–44 (106 pages). Main highlights: visit of 21 scientists who are outstanding in the international academic and scientific field. More than 3,000 participants (including researchers, University authorities, education officials, businesspeople, and others). The term *Converciencia* is used a total of 19 times in the report
2010	Rosa María Amaya	*Converciencia* is reported on three pages 52–54 (104 pages). Main highlights: Inclusion and gender perspective are incorporated in the activity with the visit of four women scientists who disseminated information about their research abroad (no other details are provided). Participation of 2,387 people, nine visiting scientists, 35 local scientists, and the development of teleconferences allowed 447 people from different locations. References are made to the signature of various international cooperation agreements with parties in Mexico, Costa Rica, and Brazil. The term *Converciencia* is used a total of 23 times in the report
2011	Rosa María Amaya	*Converciencia* is reported on three pages 46–48 (102 pages). Main highlights: Commemoration of the 7th edition. The central theme was “Environment and Population” at the suggestion of the *RedCTI* of Guatemala, with a particular focus on food and nutritional security. This year, 11 speakers from the GSD visited Guatemala, and four foreigners from Colombia, Venezuela, and Taiwan, participated. The speakers attended conferences, professional exchanges, and meetings for potential cooperation agreements. Guatemalan scientists living in the country also participated. The program received scientific and financial support for its implementation from Cyted, the International Center for Theoretical Physics (Ictp), the Taiwan government, Intecap Guatemala, and the Mariano Galvez University. The report also highlighted that for the first time, activities took place in provinces (outside Guatemala City) with the direct participation of the scientists in Chiquimula and Quetzaltenango. A cooperation agreement between Senacyt and the Center for Theoretical Physics of Colombia in research activities was signed for researchers' technological development and training in advanced areas of natural sciences. *RedCTI* celebrated its annual Assembly to accept new members and approve the work plan 2012. The term *Converciencia* is used a total of 26 times in the report
2012		*Converciencia* is reported on three pages 40–42 (72 pages). Main highlights: a workshop on Population and Territory was organized, a computer system that stores the scientific contents in the video that were treated in the event, knowledge about biotechnology was promoted to 350 high school students, and results of research related to environmental quality and water pollution. This year, 16 members of the GSD participated as speakers. They exchanged experiences and knowledge with the academic community and the public. Also, 55 scientists residing in Guatemala participated in an event organized by the Concyt Technical Commission. Four scientists from Argentina, Mexico, and Taiwan participated in the events. A set of recommendations was compiled to address national actions for the country's development. The two main topics analyzed were food and nutritional security and the country's environmental situation. In total, 5,127 people participated as an audience of *Converciencia*. Additionally, 408 connected online. A total of 62 conferences. The First Central American Workshop on Space Systems Engineering, with the participation of 25 participants, was held. The term *Converciencia* is used a total of 25 times in the report
2013	Maria Rubio Contreras	There are no references to *Converciencia* this year as the activity was suspended from 2005 to 2012. The most related event was the International Congress of Science, Technology, and Innovation; however, the specific component of including Guatemalan scientists residing abroad was eliminated. The report is 53 pages
2014	Armando PokusYaquián	The term *Converciencia* is mentioned two times on page 27 of the report; those are references to past editions of the activity. This year the activity was suspended in the way it was implemented from 2005 to 2012. The document reports the second edition of the International Congress of Science, Technology, and Innovation. Once again, no attention is paid to the participation of Guatemalan scientists residing abroad. The report is 49 pages
2015	Armando PokusYaquián	There are no references to *Converciencia* this year as the activity was suspended from 2005 to 2012. The document reports the second edition of the International Congress of Science, Technology, and Innovation. No reference to the participation of Guatemalan scientists residing abroad. The report is 35 pages
2016	Armando PokusYaquián/Oscar Cóbar Pinto	Senacyt did not release an Annual Report for the 2016 year individually; however, there is a multi-year 2016–2019 Report issued by Senacyt. It is indicated that this year *Converciencia* was suspended in the way it was implemented from 2005 to 2012. This multi-year report on page 152: “Senacyt, in 2017, relaunched *Converciencia* as the most prominent scientific outreach event in Guatemala, to promote the exchange between visiting Guatemalan scientists and their resident peers in the country, to share their experiences, present the results of their studies and delve into the current state of science in the region. It also seeks to foster a scientific and research culture in elementary, middle, and high school students. This academic activity stimulates the dissemination, promotion, and popularization of scientific and technological production through different mechanisms and methodologies, ensuring that the same reaches all audiences and actors linked to national socio-economic development.” 2016 also marked a transition from Secretary Armando PokusYanquián to Secretary Oscar Cobar Pinto
2017	Oscar Cóbar Pinto	*Converciencia* is reported on page 22 (50 pages). Main highlights: *Converciencia* is relaunched, with 20 visiting Guatemalan scientists and 4,405 attendants. Special activities were dedicated to the participation of women in science in *Converciencia*. The term *Converciencia* is used a total of nine times in the report
2018	Oscar Cobar Pinto	*Converciencia* is reported on page 25 (53 pages). Main highlights: Commemoration of the 10th edition of *Converciencia* and its contributions to the exchange of knowledge between outstanding Guatemalan scientists residing abroad with researchers, community-academic, and the public back in Guatemala; participation of 28 Guatemalan scientists in this edition in the search for links between the academic community and scientists from the public, private, and academic sectors. The event took place in Guatemala City (capital) and various provinces. The term *Converciencia* is used a total of eight times in the report
2019	Oscar Cobar Pinto	Senacyt did not release an Annual Report for the 2016 year individually; however, there is a multi-year 2016-2019 Report issued by Senacyt in which it is indicated that *Converciencia* activities took place in 16 locations within the frame of the Sustainable Development Goals of the United Nations, these being: Energy, Water, Education, Environment, Climate Change and Sustainability, Health, Food Safety, Digital Society, and Inclusion. Activities included 78 conferences, three forums, five workshops, and 12 general activities with 31 participating scientists and 5,250 people in the Capital City and other provinces. The term *Converciencia* is mentioned 18 times in this multi-year report. The report is 259 pages
2020	Ana Chan Orantes	*Converciencia* is reported on page 9 (21 pages). Main highlights: Celebration of the 12th edition. This year, the objective was to exchange knowledge between society and Guatemalan scientists who work in scientific research, technological development, and innovation institutions inside and outside the country. Main activities responded to the Ibero-American Citizen Agenda of the Ibero-American General Secretariat -Segib- and the UN Sustainable Development Goals (SDG) (Energy, Education, Environment, Climate Change, Health, Food Security, and Digital Society). *Converciencia*, for the first time, was entirely held virtually, having the participation of 37 scientists and 2,729 attendees; 39 activities were carried out. The term *Converciencia* is mentioned a total of six times in this report

*Source: own elaboration based on SENACYT (2022a,b)*.

#### The Scientific Community and the International Network of Science, Technology, and Innovation of Guatemala *(RedCTI)*

In 2005, with the framework of the first *Converciencia, RedCTI* was founded. This was promoted by the National Secretary of Science and Technology, the Presidential Commissioner for Science and Technology, eleven members of the SD of Guatemala, and eight scientists residing in Guatemala representatives of the Council of Notables (awardees of the National Medal of Science and Technology, the highest recognition for scientific work in Guatemala). The main objective of this network was to generate academic coordination between different Guatemalan institutions and to engage Guatemalan scientists with *RedCTI*. Also, part of its goals was to identify academic content of diffusion, teaching, research, innovation, science communication, and knowledge transfer for the development of Guatemala. *RedCTI* constitutes one of the main actions supported by Senacyt to promote S&T for national development. As of February 2022, *RedCTI*^2^ had 196 members. Applying a binary gender filter (male/female) in this network, 71.43% (140) are men, and 28.57% are women (56). According to their field of knowledge, this network includes 23.46% (46) in medical sciences, 31.6% (62) in natural sciences, 27.55% (54) in engineering and technology, 11.22% (22) in social sciences, and 6.8% (12) in agricultural sciences. In terms of level of education among its members, 5.1% (10) reported having completed a bachelor's degree (*licentiate*), 29% (57) master's degree, and 65.9% (129) hold doctoral degrees (Ph.D.). The reported country of residence shows that 54.6% (107) indicated their country of residence as Guatemala and that 45.4% (89) reside abroad. In this aspect, a more comprehensive concentration of GSD is found in the United States with 18.3% (36), followed by Mexico at 5.6% (11) and 4.6% in France (9). Other destinations with less presence include Argentina, Chile, Canada, Germany, England, the United Kingdom, South Korea, Taiwan, Japan, etc.

Each year, members of *RedCTI* were invited to participate in *Converciencia*. Likewise, within the framework of the event, until 2019, participation was in-person and required mobility. Members of *RedCTI* held a general assembly in which they declared the importance of the continuity of *Converciencia*. *RedCTI* considered *Converciencia* an essential space for the Guatemalan scientific community and their interaction with society, sharing the country's scientific developments.

#### The Host Institutions

Since the first edition of *Converciencia*, the role of the host institution was designed as a co-organizer sharing with Senacyt logistic, organizational, and follow-up responsibilities. The level of involvement of each host institution and the number of participants (single or recurrent) have changed over time. Universities, research centers, and a few public institutions have played a recurring role in co-organizing *Converciencia*. Among their functions, host institutions provide infrastructure (their facilities host the activities) and audience (professors, researchers, students, and staff). The activities, in the context of *Converciencia*, include academic events (round tables, panels, and discussions by experts) and visits to laboratories and different facilities. Host institutions have expressed their intention to have more influence on planning and organizing *Converciencia*. In other words, they seek to act as true co-organizers and not as passive bystanders. However, as it will be discussed later, their involvement has been limited to logistics (receiving guest speakers, attending technical aspects of the events, and providing transport to participants), with no influence on the organizational aspects of the event.

#### Communication Agents and the Local Media

Senacyt has reached out to the media to provide coverage for *Converciencia* activities and speakers. The coverage has been focused on highlighting the academic background of the speakers, the activities in the context of *Converciencia*, and the roles and contributions of the host institutions, especially in cases with interactions between scientists, children, and young students. Frequently, the coverage has transcended the informative perspective incorporating a critical or reflective approach. News and reports have highlighted the importance of increased investment in science, technology, and innovation for Guatemala. Also, media posts written by scientists living in Guatemala have described the need to rethink *Converciencia* toward GSD engagement with local scientists beyond dissemination activities.

Reviewing the news coverage and publications addressing *Converciencia* in the leading national newspapers (see Table 7) makes it possible to extract headlines and trends in the program's coverage from 2005 to 2020. The review was conducted entirely online, representing a limitation as it was not a comprehensive hemerographic inquiry. Particularly in the early editions of *Converciencia*, the penetration of the internet was limited in Guatemala, and media coverage was mainly done on printed (paper) means. Notwithstanding, the reviewed media coverage included three private newspapers, *Prensa Libre, El Periódico*, and *La Hora, one* public newspaper, *Diario de America Central*, and one digital portal, *Soy502*. These news reports were the ones found in online search engines using the keyword “*Converciencia* Guatemala.”

**Table 7:** Descriptive analysis: selected media coverage, *Converciencia* (2005–2020).

**Year**	**Headline/scope and content**	**Links**
2009	Science in Guatemala, notes from *Converciencia* 2009 by Fernando Quevedo	https://guateciencia.wordpress.com/2009/08/15/converciencia-2009-2/
2009	UN-Spider present in *Converciencia* 2009 UN-Spider was invited by the Guatemalan Council of Science and Technology to participate in *Converciencia*, which forms part of a strategy to promote the development of science, technology, and innovation within Guatemala	https://un-spider.org/sites/default/files/UN-SPIDER_Updates_July_2009.pdf
2011	*Converciencia* Guatemala 2011. *Converciencia* is the meeting of society with Guatemalan scientists who work in research inside and outside the country	https://ilifebelt.com/converciencia-2011/2011/07/
2012	*Converciencia* has motivated the interest of Guatemalan scientists—based abroad—in trying to collaborate with the country	https://www.prensalibre.com/guatemala/datos-satelites-ayudarian-pais_0_745725440-html/
2017	*Converciencia* 2017 promotes development through science and technology. The meeting of scientists is a space for the exchange of ideas and works based on science in which 26 Guatemalan scientists who work inside and outside the country will participate	https://www.prensalibre.com/tema/converciencia-2017/
2018	*Converciencia* 2018 will bring together scientists from around the world. Guatemala will bring together a quarter of a hundred scientists at *Converciencia* 2018. The event's motto will be “10 years believing in the brilliant minds of Guatemala”	https://republica.gt/2018/07/13/se-viene-converciencia-2018/
2018	Science as an escape route from underdevelopment *Converciencia* celebrates 10 years as a meeting point for national scientists residing in Guatemala and abroad and 10 years of opening a window to the development of science	https://nomada.gt/blogs/la-ciencia-como-ruta-de-escape-del-subdesarrollo/
2018	*Converciencia* as a plan to prevent brain drain. Nearly 800 Guatemalans have a doctorate, most of them obtained it abroad, and where they studied, they have stayed to live. Senacyt thought of creating a system that would offer a space for these scientists to return and share academically what has been learned outside the borders.	https://www.soy502.com/articulo/senacyt-tiene-plan-evitar-fuga-cerebros-24039
2018	Meet the brilliant minds of Guatemala. Enrique Pazos and Eduardo Rubio, physicist and astrophysicist, respectively	https://www.soy502.com/articulo/conoce-mentes-brillantes-guatemala-149
2019	Thirty-two national scientists shared experiences in *Converciencia*. Of these, 25 work abroad and 7 in Guatemala. It is estimated that 8 thousand people will participate in the scientific conclave, which will address issues on energy, education, health, food security, inclusion, environment, water, and others	https://dca.gob.gt/noticias-guatemala-diario-centro-america/32-cientificos-comparten-su-experiencia-en-converciencia/
2020	15 years of the existence of *Converciencia*. Juan Diego Chang described how the first events of *Converciencia* motivated him to study physics. Nevertheless, his opinion is that the event needs to evolve after more than 15 years. Bringing top-level Guatemalan scientists once a year is helpful, but not exclusively to give talks to the public	https://www.plazapublica.com.gt/content/quince-anos-de-converciencia
2020	Science was being carried out in Guatemala despite indifference. Local scientists described how investment in scientific research in Guatemala is scarce and believed that *Converciencia* has not produced the effect that Guatemalan scientists need	https://elperiodico.com.gt/noticias/domingo/2020/09/06/hacer-ciencia-en-guatemala-a-pesar-de-la-indiferencia/
2020	Challenges for science in Guatemala. Critical scientific advances are presented in the country, but there is still a gap in covering the entire population's needs	https://www.prensalibre.com/vida/escenario/retos-para-la-ciencia-en-guatemala/
2020	Cristina Domínguez, a Guatemalan participant in *Converciencia*, investigates how to help communities worldwide where electricity is a dream	https://www.prensalibre.com/revista-d/guatemalteca-investiga-como-ayudar-a-comunidades-del-mundo-donde-la-energia-electrica-es-un-sueno

*Source: own elaboration based on media review*.

### Explanatory Analysis

This level of analysis refers to the argumentation regarding elements that impact or cause the analyzed policy/program/practice.

**Figure 2:**
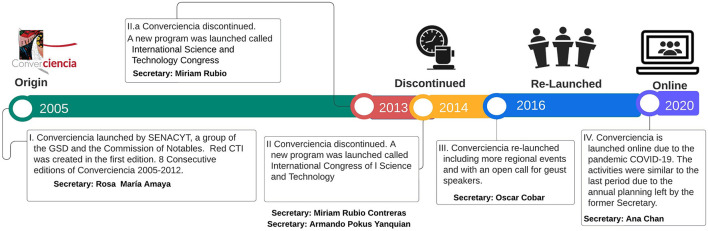
Timeline: *Converciencia* (2005–2020). Source: own elaboration.

As indicated before, Senacyt is responsible for planning and coordinating *Converciencia*. The activities related to this practice have changed, reflecting the vision and priorities set by the institution's top authority. Figure 2 illustrates the timeline of *Converciencia's* key events. The following analysis was developed according to interviews with each National Secretariat of Science, interviews with host institutions, and public report results from the annual activities of Senacyt. In countries such as Guatemala, where re-election is impossible, governmental officials may show a terminal logical behavior and strategic defection during transitions that affect policy sustainability. Also, new government officials dissociate from the outgoing government to fit in with the new government, leading to a lack of continuity (Escobar-Alegria et al., 2020).

#### Effects of Policy Shifts in *Converciencia*

Guatemala's national S & T system is a complex array of linkages between institutional representatives from entities in the public sector, the academic sector, and the private sector. Concyt governs this system (Congreso de la República de Guatemala, 1991). Concyt is integrated by nine members and is chaired by the country's vice-president. Concyt is the decision-making body providing orientation and direction to the country's scientific and technological policies and guidelines. The institution implementing such a vision is the National Secretariat of Science and Technology (Senacyt). In other words, the top authority who can influence the STI policy shifts in Guatemala is the Vice President. In this sense, at this level of analysis, it is relevant to have in perspective who has occupied such positions during the study period.

#### *Converciencia* 2005–2012 (Vice President Eduardo Stein From 2004 to 2008 and Vice President Rafael Espada From 2008 to 2012)

Secretary Rosa Maria Amaya was among the co-founders of *Converciencia* in 2005. Her background career is in economics and project management. Her professional trajectory has progressed primarily in public administration and academia. Her linkages with scholars and researchers were strong. She purposefully built ties with the Presidential Commissioner of Science and Technology, most prominent Guatemalan researchers, and GSD members, which allowed her to launch the first *Converciencia*. The main objectives of this initiative were (i) strengthening science and technology in the country, (ii) exchanging experiences with the GSD, and (iii) seeking solutions to latent problems in Guatemalan society.

Additionally, to be the first program of its kind searching for collaboration with GSD and local scientists, the results from the first *Converciencia* went beyond founding *RedCTI*. Not all established local researchers participated in the conception of *Converciencia*, only awardees of the National Science Medal. This created continuous friction over time. Various researchers working and residing in Guatemala with established careers describe *Converciencia* as an elitist event where they were not invited, and their work in the country was not valued.

#### *Converciencia* 2013–2016 (Vice President Roxana Baldetti From 2013 to 2015 and Vice Presidents Alejandro Maldonado and Alfonso Fuentes, 2016)

In the following period, under the mandate of Secretary Miriam Rubio Contreras (January 2013–September 2014), with a background in industrial engineering and academic and industry trajectory, *Converciencia* was suspended (Cobar, 2015). Rubio Contreras indicated she “could not identify the social impact of *Converciencia* after reviewing the investment budgets in the previous years.” Therefore, Rubio Contreras decided to launch the International Science and Technology Congress, with similar activities as *Converciencia* but opening other spaces for the *RedCTI* interaction with local researchers. According to her interview, a program of interchange with the former International Coordinator of *RedCTI* opened exchange programs with several students. Local researchers identified these events as having similarities to *Converciencia*. However, scientists from the Guatemala diaspora were not purposefully invited.

The National Secretary of Science, Armando Pokus Yanquian, with a background in civil engineering and project management, considered that *Converciencia* was not valuable. He did not identify a positive impact of bringing the scientists' diaspora to Guatemala for one week a year to Guatemala City to share experiences, especially when 80% of the participants were the same each year. Pokus Yanquian launched the National Week of Science and Technology in different regional locations of the country. His goal was to engage the youth to pursue science careers in other departments of Guatemala. Local researchers liked the regional approach, but the program was focused on dissemination to young generations and there were no other potential collaborations. The GSD was not invited to this event, and *RedCTI* was inactive. This period shows a steep decrease in the participation of scientists. The general political situation in Guatemala was unstable, reflected in the consecutive transition between three vice presidents in this period. Cobar (2015) reported:

The dissemination of scientific advances in the country and the world are discussed at the Annual Conference on Science, Technology, and Innovation. This event has been held since 2013 in Guatemala City and several departments inside the country. The Congress is attended by national and international scientists who are experts in various branches of knowledge. The investigations carried out in the system are disclosed, forum panels are held, and seminars on current issues of national interest are conducted, among others. This event replaces *Converciencia* and the National Science and Technology Week events held previously (Cobar, 2015, p. 191, 192).

#### *Converciencia* 2017–2019 (Vice President Jafeth Cabrera)

Oscar Cobar Pinto was the Secretary who relaunched *Converciencia* after the political period between 2013 and 2016. Cobar's background is in organic chemistry; he is part of the GSD that came back to the country. He was awarded the National Medal of Science in 2002; he was a member of the Notable Commission of Guatemala and a *Converciencia* co-founder. His strong relationships with the Guatemala System of Science and Technology (Sincyt) led him to envision linking the GSD with the scientific community in the country. He was interested in bridging the academic sectors from Guatemala with the GSD for potential collaborations. In addition, his priority was to promote science for the Guatemalan new generations. One of the first modifications of *Converciencia* during that period was to locate some activities in different regions of the country and different host institutions using the universities as part of the scenarios to develop collaborations. *Converciencia* had public open calls to apply as a speaker, making it easier for other scientists interested in the event. Host institutions selected their preferred speaker and activity from a list provided by Senacyt. The GSD participating in *Converciencia* felt a significant affinity to the vision of the head (related to his scientific background). Most of the scientists from the diaspora felt that their voices were heard. Nevertheless, some local scientists considered they were not the protagonists of these events, causing conflicts in their perception. Host institutions also felt left out of the planning, limiting the outcomes.

#### *Converciencia* 2020 (Vice President Cesar Guillermo Castillo)

At the time of this research, Ana Judith Chan was the incumbent National Secretary of Science, who has a master's degree in International Business Law. She is a former President of the Guatemalan Mexican Chamber of Commerce and Industry (Camex). She considered *Converciencia* as “one of 20 activities implemented by Senacyt each year.” Her vision for *Converciencia* was to restructure the activity to be more connected to the Guatemalan population, the country, and the industry's needs. She considered the past events of *Converciencia* as activity occasions disconnected from the harsh realities of Guatemala. Her perception of *Converciencia* was of a time and resource-consuming event with limited results and impact. In 2020, *Converciencia* was developed without significant changes in organization and scope; however, due to the COVID-19 pandemic, the event was entirely implemented through remote/digital online formats with no in-presence activities. Researchers appreciated that the event was online, and there were no significant expenses involved (mobility, air travel, logistics). The GSD participating in the event had a larger audience, but interactions with peers and networking opportunities were constrained.

### Normative Analysis

This level of analysis refers to the set of ideas and the worldview held by the actors that influence (justify) their behavior, actions, and decisions. It is based primarily on their interpretation of the ideas that shape the policy/program/practice under analysis.

#### *Converciencia* Limitations

**Figure 3:**
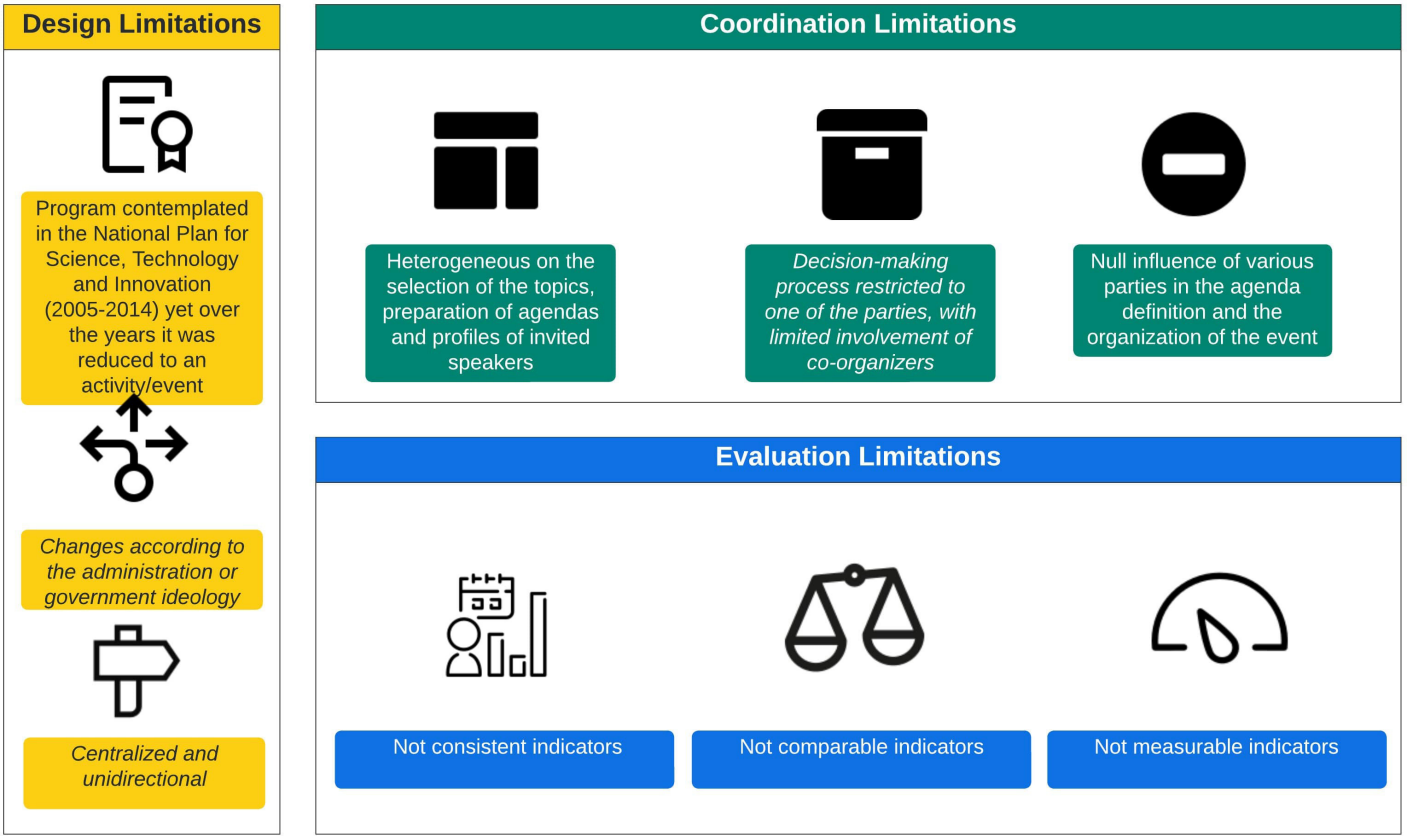
Limitations of *Converciencia* as a science and technology practice. Source: own elaboration.

The analysis of Senacyt's annual reports released between 2005 and 2020, contrasted with the assessments derived from the focus groups, allows for the emergence of *Converciencia's* limitations found in its design, coordination, and evaluation processes. These three aspects show that although Senacyt gave greater relevance to the participation of the scientific diaspora during the days of the event, they were neither included in the previous stages (planning) nor in the posterior stages (monitoring, evaluation). The priorities of *Converciencia* topics were driven by the government's vision and did not engage the GSD with the rest of the scientific system. Figure 3 illustrates *Converciencia* limitations.

##### Design Limitations

Although *Converciencia* was conceived as a program contemplated in the National Plan for Science, Technology, and Innovation (2005–2014), over time, the policy perspective shifted and reduced it to a yearly event. The annual reports repeated knowledge exchange as a critical characteristic of *Converciencia*, but according to Participant (2022b), the dissemination activities were from the GSD to the participants with a unidirectional approach. Also, *Converciencia* has a centralized approach because it has been held mainly in the capital city^3^. Despite the event being sustained over time, the general objective has not been standardized and has changed confusingly and diffusely in each new administration.

##### Coordination Limitations

There is no homogeneous process and clear criteria regarding who and how the topics of discussion for each event are defined. The decision-making process is restricted to Senacyt in various editions of *Converciencia*. The institutions have not involved the scientific community represented by *RedCTI* in the organization, elaboration of a plan, and activities, even though this network of Guatemalan scientists was created precisely as the co-founding party *Converciencia* back in 2005. There is no specific information on whether the decisions^4^ for the scope and thematic to be included respond to the agenda of the government, the scientific community^5^ (or the network), the international community, civil society, or the national scientific and technological system. This reveals the reduced or even null participation of other sectors in the definition of the agenda and its priorities and the general organization of the event, which affects the effectiveness, willingness, and viability of the dissemination and exchange of knowledge with the GSD.

##### Evaluation Limits

The annual reports from the last 15 years have not included any information about financial resources and the budget allocated to *Converciencia*. Moreover, the results and impact indicators are not comparable between years. For example, only in a few editions, the number of academic collaborations between researchers was measured. The reports focused on the number of speakers and participants. However, no specific information on the type of knowledge exchange, agreements developed, joint publications, and different collaboration categories has been reported.

#### *Converciencia* SWOT Analysis

**Figure 4:**
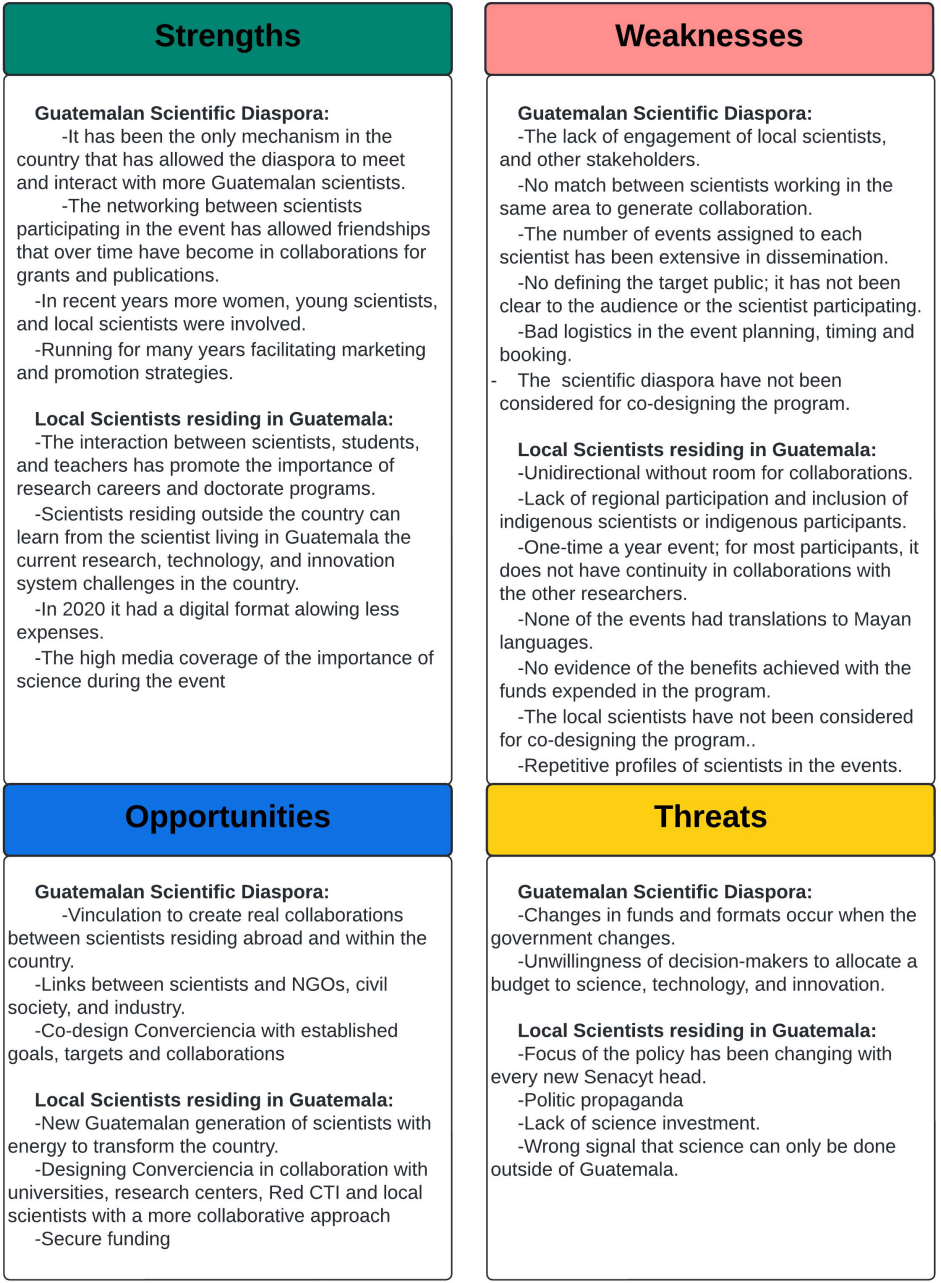
*Converciencia* strengths, weaknesses, opportunities, and threats *(*SWOT) analysis. Source: Own elaboration.

To better understand the perception of the scientists who have participated in past editions of *Converciencia*, a SWOT analysis was conducted with data derived from the focus group discussions and interviews with 25 scientists and 3 stakeholders representing civil society, industry, and academics (see Figure 4).

##### Strengths

*Converciencia* has enabled interaction between scientists from the diaspora and those working in the national territory with students, educators, and broader sectors of Guatemalan society. The event has shown undergraduate students the opportunity to become researchers and pursue graduate studies, primarily to obtain a doctoral degree. In recent years, scientists who have participated in *Converciencia* have identified the program's transformation involving more women, young scientists, and local scientists as speakers. Also, in the last years, more locations have been included, decentralized to the capital city, and even involved a digital format in 2020 because of the COVID-19 pandemic. Another advantage is that scientists outside the country can learn the current research, technology, and innovation system challenges from scientists living in Guatemala. In addition, scientists from the GSD identify that one of the main strengths of *Converciencia* is that it has been the only mechanism in the country that has allowed them to meet and interact with more Guatemalan scientists. The networking between scientists participating in the event has allowed for friendships that have become collaborations for grants and publications over time. Due to the country's conditions, scientists conducting research work in Guatemala engage in collaborative work with scientists residing in other countries who participate in *Converciencia* with their own institutional or personal funds (no receiving financial support from a Guatemalan institution/organization). Researchers appreciate the GSD attitude toward learning and collaboration. Media coverage of the importance of science during the event has been one of the strengths highlighted by local researchers. Moreover, *Converciencia* is well-established and well-known, and has been running for many years, facilitating marketing and promotion strategies. The joint doctoral program between Unam and Usac organized by one of the researchers visiting the event has been one of the outcomes of *Converciencia* that have generated measurable results, i.e., various Guatemalans who graduated from doctoral programs currently occupy leading positions in academia, mobility, and research exchange between Unam and Usac, and other collaboration agreements with Cambridge University and Ictp organized by the other co-founders of *Converciencia*.

##### Weaknesses

Lack of engagement of local scientists with the event has been identified in all the interviews. There have not been enough incentives for local scientists to participate. There has not been a match among scientists working in the same area to generate spaces for collaboration. The number of events assigned to each scientist has been extensive, limiting only to disseminating research results and not allowing communication between different sectors to evaluate collaboration opportunities. *Converciencia* is a one-time a year event; for most participants, it does not have continuity in collaborations with other researchers. Many activities have been organized without defining the target public; it has not been clear to the audience or the scientists participating. Stakeholders from the industry had identified the lack of participation of industry and other sectors in these events. Also, the profiles of scientists have been repetitive every year. Participants have also specified bad logistics in event planning, timing, and booking, including lack of regional participation and inclusion of indigenous scientists or indigenous participants. None of the events were available in indigenous languages. Most of the researchers residing in Guatemala consider that the program has more weaknesses than strengths. They have described the program as unidirectional without room for collaboration. Additionally, there is no evidence of the benefits achieved with the funds expended in the program. Guatemalan scientists, including the members of the *RedCTI*, have not been considered for co-designing the program. This group should be fundamental to *Converciencia* planning due to their seminal contribution to the origin of the program.

##### Opportunities

One of the main opportunities identified is that the platform could allow for follow-up and concrete collaborations between scientists residing abroad and in the country. It can also promote connections among scientists, NGOs, civil society, and industries. Also, a new cohort of scientists has been identified as an opportunity. This is because of the renewed energy and innovative ideas that they can potentially bring. Designing *Converciencia* in collaboration with universities, research centers, *RedCTI*, and local scientists has been identified as an opportunity to create longer and established relationships between the participating scientists. According to the local researchers, The *Converciencia* funds' allocation in the Senacyt annual operation plan should continue but under a collaborative approach focusing on Guatemalan researchers' needs. *Converciencia* has an assigned annual budget that can continue over time and is an advantage to consider with a better restructuring and with a more collaborative approach to listening to the Guatemalan researcher's needs. Likewise, there is an opportunity to invite scientists who carry out scientific dissemination to participate in the event.

##### Threats

The main threats involve changes in policy direction, budget allocation, and structure of the event which occur when the government administration changes. The focus of *Converciencia* has been changing with every transition in the top direction of Senacyt. Moreover, there is not enough participation of established local scientists who consider *Converciencia* an elitist event. They perceive that the event only highlights the work of Guatemalan scientists residing abroad, along with local results. Another threat identified by researchers living in Guatemala is the potential message that science can only be done outside Guatemala. According to researchers, politicians on duty have been another threat since they guide the event for their propaganda interests. The most significant danger mentioned by all scientists is the unwillingness of decision-makers to allocate a budget to science, technology, and innovation.

**Figure 5:**
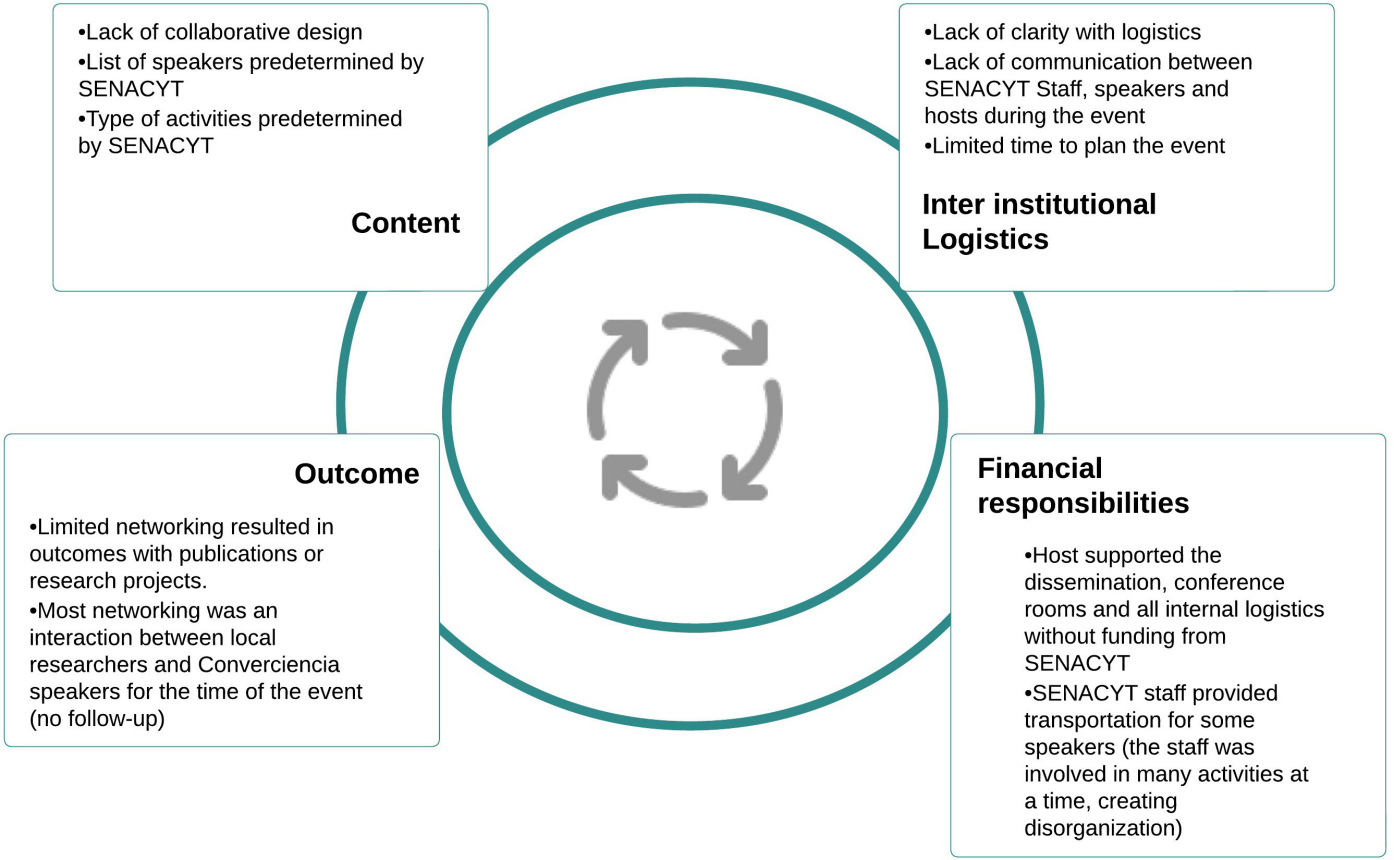
Co-organizing *Converciencia*: perspective from the host institutions. Source: own elaboration.

The perspective of the host institutions is critical for explaining different challenges and opportunities experienced in their roles as co-organizers of *Converciencia*. Figure 5 summarizes the main findings derived from the data collected from the institutional actors.

### Prescriptive Analysis

This level of analysis refers to the “ideal” version of the policy/program/practice, for which actors and stakeholders present a set of recommendations to improve.

#### *Converciencia* As a Policy and Practice

The evidence of successful results from *Converciencia* offers opportunities to assess its relevance as a policy/practice. Participant (2022b) provided a few examples of mobility and international collaborations rooted in the first edition of *Converciencia*; “various outcomes can be traced back to *Converciencia* including the creation of a “joint doctoral program” co-organized and implemented between the University of San Carlos of Guatemala and the Autonomous University of Mexico.” Another example is the research visits of Guatemalan students to the International Center for Theoretical Physics (Ictp) in Italy. In addition, the transfer of environmental virology with the University of Barcelona was promoted. These three initiatives were promoted by three scientists from the GSD who co-founded *Converciencia*, Concepción Toriello, Fernando Quevedo, and Oscar Cobar Pinto. These examples shared as a common feature the need to follow up and engage in sustained collaborations as opposed to episodic, isolated activities.

According to Participant (2022a), a vision focused on entrepreneurship has been favored instead of research and development in recent years, even in the health crisis derived from the COVID-19 pandemic. Participant (2022a,b) considered that *Converciencia* should focus on inter-institutional exchanges and transfer knowledge by co-creating postgraduate programs with GSD institutions and local institutions. Another participant provided an example of results from *Converciencia*, a joint publication derived from the 2019 edition in which a member of the GSD working in Chile collaborated with scholars in the host institution and presented the research results in international conferences and journals.

*Converciencia* has responded, according to some comments from the Participant (2022b), to an elitist vision resulting in exclusionary practices. These cognitive characteristics in public management generate biases that privilege the “well-known” [speaker's profiles] and devalue diversity. *Converciencia'*s repeated patterns of lack of institutionalized policies are due to the volatility and distrust from Senacyt and/or Concyt top authorities^6^. These patterns relegated the local scientists and the GSD in the decision-making, management, and engagement.

*Converciencia's* strengths and weaknesses over the years can lead us to restructure policies according to the demands of the scientific community and social and economic needs. Nevertheless, *Converciencia* has a strong policy to engage the GSD, and local scientists need a co-governance model with a stable budget. Moreover, Senacyt and Concyt need to plan beyond administration and focus on the scientific content from a collaborative perspective with the key actors. These collaborations will create an environment for brain re-circulation and diaspora networking.

#### *Converciencia* As a Potential Successful GSD Engagement Policy

All the actors involved in different periods and stages have highlighted several weaknesses of this policy. Nevertheless, it does not mean that the scientific community is not interested in the continuation of the program. The Guatemalan scientific diaspora and local Guatemalan researchers agree that *Converciencia* must continue with restructuring and co-design and with the participation of the Guatemalan scientific community and other key stakeholders. There is a growing interest in the application of “design thinking” to policy-making (McGann et al., 2018). In the following sections, we describe five critical steps of co-design thinking that could be adapted to the policy and potential recommendations for this process. Usually, government science policy organizations in developing countries do not coordinate their activities between possible users of their technological innovations that are absent or unsatisfactory (Crane, 1977). The development policy is often considered policy creation found in advanced economies and developing countries' interventions and should be limited to promotion (Bell and Hindmoor, 2009). Accordingly, there is a democratic deficit of citizen participation in the definition and formulation of issues concerning science, technology, and innovation, contrary to the strong involvement of entrepreneurs and corporations (Viales-Hurtado et al., 2021).

##### Empathizing With the GSD and Scientific Community Back in Guatemala

According to recent research in science policy practices for Guatemala, some key and charismatic individuals can act as door openers to link community organizations and science policy networks (Aguilar-Støen, 2018). Engagement by focus group discussions or online surveys could help Senacyt to understand the view of the parties involved by incorporating different perspectives. It can help understand the perspective of local scientists and their need to collaborate with the SD including scientific dissemination, research exchange programs, co-writing research proposals for international and national funding, and opportunities for equipment donation. It can help understand the perspective of the SD and their readiness to contribute to strengthening the S&T context of their country of origin. The GSD can collaborate with local researchers with respect to sharing and learning scientific knowledge, available funds for supporting research, potential collaboration agreements with their affiliated institution and Guatemalan institutions for joint research, channel donations, grants, and scholars' opportunities among others. It can help understand the perspective of universities and their need for scientific knowledge, scholarship for students, researchers, and professors, among needs for co-designing doctorate and masters' programs with a mobility format. There is evidence that the role of universities in Central America as research and innovation hubs could support design and implement flexible, transparent, and robust strategies toward the achievement of sustainability in the region. The thematic, technical, evaluative, and procedural areas provide a comprehensive framework to build capacities adapted to the functions and responsibilities of the actors (Miquelajauregui et al., 2021). It can also help understand the perspective of research centers and their need for equipment, funds, research interchanges, and co-teaching. This can also help understand the perspective of the industry and their need for the technology solution and research and development to innovate their products or services. It can help understand the perspective of civil society organizations and their needs to solve community and country health, economic, nutrition, energy, and other critical problems. Finally, it can help understand the perspective of the government and their need to support their work with science and technology, i.e., the Ministry of Health and their management of the pandemic and the Ministry of Education and their STEM programs.

Recent research on multi-level storylines applied to socioeconomic and hydrological processes showed results of engagement with the indigenous Mayan community from Atitlán (Bou Nassar et al., 2021) (i) helped develop an understanding of scientific mechanisms, (ii) initiated a dialogue between indigenous people and non-indigenous stakeholders, and (iii) extracted potential solutions targeting the system's leverage points. Therefore, this methodology could be helpful for *Converciencia* policy planning involving marginalized stakeholders, mainly Mayan communities. Strengthening the roots, increasing collaboration, and capacity-building are undoubtedly central to the bottom-up theory for science, technology, and innovation activities (Lema et al., 2021).

##### Defining Strategic Collaborations

There are plenty of problems in Guatemala including: quality of education, poverty, food insecurity, weak governance, endemic corruption, violence, citizen insecurity, lack of respect for human rights, and inequitable access to economic opportunities (U.S. Department of State, 2021). The National Plan of Development K'atun prioritizes the progress of a rural and urban Guatemala, wellbeing, present and future use of natural resources, and the role of the state as guarantor of human rights and driver of development. The National Policy of Science and Technology 2015–2022 prioritizes forming high-level human capital, research based on social and productive demands, innovation and technology transfer, and scientific and technological popularization. Therefore, linking the ideas collected from the empathize stage with the lines of priority for the country defines the problem to be solved in *Converciencia* as a policy and not only as an event. Nevertheless, none of these recommendations could work without the commitment of key actors, including the *RedCTI* created during the first year of *Converciencia*. Also, Senacyt should be committed to courses of action and funding priorities concerning *Converciencia* as an engagement policy with the GSD.

##### Ideating With the Key Actors

Ideating is the process of thinking outside the box by brainstorming with key actors to attack the problems defined. This stage aims to design a solution for restructuring *Converciencia policy*. Citizens, local researchers, and the GSD may suggest relevant local knowledge and contribute novel ideas since they are not burdened by professional expertise (Reich, 1996; Fung, 2006). Ideating is also an interactive stage between prototyping and testing.

##### Prototyping the Solution

The policy cycle model has a sequential development rational approach. The model first defines the problem, then formulates the policy and implements it, and finally evaluates it (Knill and Tosun, 2020). Usually, in this structure, the decision-making on the final policy is focused on the policy sphere and not on the key actors. Prototyping has a more humanistic approach to the policy than a systemic, deterministic approach (Camburn et al., 2017). Prototyping is learning about an idea's strengths and weaknesses and identifying new directions (Brown, 2008). This stage is not focused on the outcome but on the learning that helps policymakers, researchers, and key actors make the policy's idea more real before investing in it (Stanford Law School, 2018). The role inspires a taxonomy for policy prototypes, look and feel, and implementation. In this case, the Senacyt team, *RedCTI*, researchers, and other actors interested in the codesigned policy could analyze the following questions: (i) How does *Converciencia* policy impact the GSD? (ii) How does it feel to the GSD, local researchers, and the research, technology, and innovation system representatives? (iii) How does it work for the GSD and local researchers?

##### Testing *Converciencia* as an Engagement Policy

**Figure 6:**
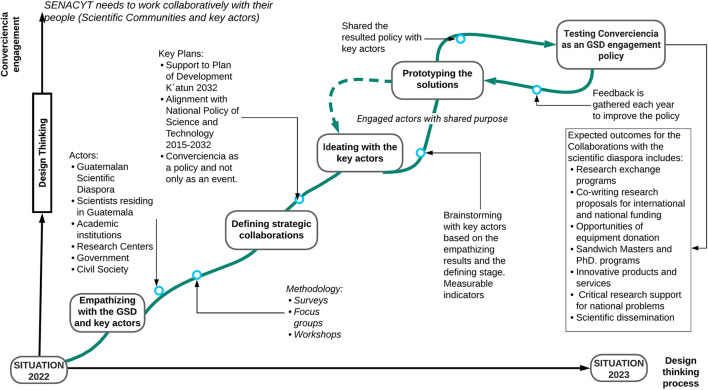
*Converciencia* as a policy to engage the Guatemala scientific diaspora (GSD). Source: own elaboration.

The *Converciencia* policy is implemented during the first year, and feedback from key actors is gathered. The iterative effect supports an improvement of the policy. For this stage, it is vital to have key indicators and metrics that measure the main results' progress over time. Until recently, the indicators presented are (i) the number of dissemination activities, (ii) the number of participants, and (iii) the number of speakers. Nevertheless, some of the expected indicators from scientists and host institutions for an efficient *Converciencia* policy are: (i) the number of research visits from local Guatemalan researchers, professors, and students to foreign GSD affiliated institutions, (ii) the number of exchange research visits from the GSD to Guatemala, (iii) the number of co-written research proposals between the GSD and local researchers, (iv) the number of scientific publications that resulted from collaborations between *Converciencia* participants, (v) the number of scholarships for local students, professor, or researchers that resulted from interaction with the GSD, (vi) the number of equipment donated through the GSD management, (vii) the number of joint Master or Ph.D. programs co-created with the GSD and local scientists, (viii) the number of young Guatemalan inspired to pursue a scientific career due *Converciencia*, (ix) the number of research or development projects funded and approved because of the collaboration of *Converciencia* participants, (x) the amount of investment from the Guatemalan industry for research projects with *Converciencia* participants, and (xi) the number of new spin-offs or start-ups generated through research results of *Converciencia* collaborations, among others. Figure 6 illustrates using design thinking for the prescriptive analysis of the ideal *Converciencia*.

## Author Contributions

KB: conceptualization (lead), data curation (lead), project administration (lead), methodology (lead), resources (lead), validation (lead), and writing the original draft (lead). SA: conceptualization (equal), data curation (equal), supervision (equal), project administration (equal), writing the original draft (equal), and visualization (lead). LGVP: investigation (supporting), visualization (supporting), conceptualization (substantial), and writing (original draft, supporting). All the authors contributed to the article and approved the submitted version.

## Conflict of Interest

SA was employed by New Sun Road. The remaining authors declare that the research was conducted in the absence of any commercial or financial relationships that could be construed as a potential conflict of interest.

## Publisher's Note

All claims expressed in this article are solely those of the authors and do not necessarily represent those of their affiliated organizations, or those of the publisher, the editors and the reviewers. Any product that may be evaluated in this article, or claim that may be made by its manufacturer, is not guaranteed or endorsed by the publisher.

## Footnotes

^1^See https://www.senacyt.gob.gt/.

^2^See https://RedCTI.senacyt.gob.gt/portal/index.php/investigadores/directoriocti.

^3^There were some efforts to extend the event to the countryside, as in the 2011, 2018, 2019, and 2020 editions.

^4^Except for the 2011 edition where that year's report mentions that the central theme “Environment and Population” was chosen based on the Network's suggestion.

^5^Although the reports make direct reference to the fact that issues addressed are of national interest, and in the case of the 2019 and 2020 editions, arise from the Citizens' Agenda in Ibero-America and the Sustainable Development Goals, they do not clarify whether the choices were adopted in coordination with the diaspora, social organizations, or the offices of the Ibero-American General Secretariat and the UN in Guatemala.

^6^Except for the Cobar administration, the Participant (2022a) stated that it has been difficult to work with the Secretaries of Senacyt, because they have had limited understanding of the complexities faced by scientists, and that they have focused merely on administrative issues with no context of scientific production.
